# Fast analysis of biobank-size data and meta-analysis using the BGLR R-package

**DOI:** 10.1093/g3journal/jkae288

**Published:** 2024-12-09

**Authors:** Paulino Pérez-Rodríguez, Gustavo de los Campos, Hao Wu, Ana I Vazquez, Kyle Jones

**Affiliations:** Colegio de Postgraduados, Montecillo, Estado de México 56230, México; Department of Epidemiology and Biostatistics, Michigan State University, East Lansing, MI 48824, USA; Institute for Quantitative Health Sciences and Engineering, Michigan State University, East Lansing, MI 48824, USA; Department of Statistics and Probability, Michigan State University, East Lansing, MI 48824, USA; Department of Epidemiology and Biostatistics, Michigan State University, East Lansing, MI 48824, USA; Department of Epidemiology and Biostatistics, Michigan State University, East Lansing, MI 48824, USA; Institute for Quantitative Health Sciences and Engineering, Michigan State University, East Lansing, MI 48824, USA; Department of Epidemiology and Biostatistics, Michigan State University, East Lansing, MI 48824, USA

**Keywords:** genomic prediction, polygenic scores, Bayesian models, meta-analysis, sufficient statistics, summary statistics

## Abstract

Analyzing human genomic data from biobanks and large-scale genetic evaluations often requires fitting models with a sample size exceeding the number of DNA markers used (n>p). For instance, developing polygenic scores for humans and genomic prediction for genetic evaluations of agricultural species may require fitting models involving a few thousand SNPs using data with hundreds of thousands of samples. In such cases, computations based on sufficient statistics are more efficient than those based on individual genotype–phenotype data. Additionally, software that admits sufficient statistics as inputs can be used to analyze data from multiple sources jointly without the need to share individual genotype–phenotype data. Therefore, we developed functionality within the BGLR R-package that generates posterior samples for Bayesian shrinkage and variable selection models from sufficient statistics. In this article, we present an overview of the new methods incorporated in the BGLR R-package, demonstrate the use of the new software through simple examples, provide several computational benchmarks, and present a real-data example using data from the UK-Biobank, All of Us, and the Hispanic Community Health Study/Study of Latinos cohort demonstrating how a joint analysis from multiple cohorts can be implemented without sharing individual genotype–phenotype data, and how a combined analysis can improve the prediction accuracy of polygenic scores for Hispanics—a group severely under-represented in genome-wide association studies data.

## Teaser

Here Pérez-Rodríguez *et al*. present a new set of computational routines for analysis of biobank size-data which for genomic prediction. Routines were added to the existing R package BGLR which will allow users to fit linear models with sufficient statistics based on genotypic and phenotypic data very efficiently from a Bayesian perspective. The authors illustrate the use of the proposed software through examples with real data.

## Introduction

Bayesian models are commonly used for the analysis and prediction of complex traits (e.g. de los Campos, Naya, *et al.* 2009; Vilhjámsson *et al.* 2015). The Bayesian repertoire of models available is extensive and includes various types of parametric models using shrinkage and variable selection priors (Gianola *et al.* 2009; Gianola 2013) as well as semi-parametric procedures (e.g. Bayesian Reproducing Kernel Hilbert Spaces regressions, RKHS, Gianola and van Kaam 2008; de los Campos, Gianola, *et al.* 2009; de los Campos *et al.* 2010).

The large set of priors developed in the last two decades offers great flexibility for modeling different genetic architectures. Importantly, unlike penalized regressions, Bayesian methods offer adequate quantification of uncertainty which is essential for any inferential task. Furthermore, Bayesian models have been shown to be competitive, and very often outperform, non-Bayesian methods (e.g. penalized regression) in prediction (Vilhjámsson *et al.* 2015; Azodi *et al.* 2019).

Among the many Bayesian shrinkage and variable selection software packages available (Pérez *et al.* 2010; Pérez and de los Campos 2014; Vilhjámsson *et al.* 2015; Cheng *et al*. 2018; Wang *et al.* 2020), BGLR (Pérez and de los Campos 2014) stands as one of the few that implements a large collection of Bayesian regression models, including seven different priors for parametric variable selection and shrinkage as well as semi-parametric procedures (RKHS, Table 1). Unlike other software packages, BGLR allows users to specify different priors (and to estimate separate hyper-parameters) for input subsets. All these features have made BGLR a popular software package for genomic analysis of complex traits.

**Table 1.** Prior distributions implemented int the BGLR and BLRXy functions.

Model	BGLR	BLRXy
Flat (FIXED)	✓	✓
Gaussian (BRR)	✓	✓
Scaled-*t* (BayesA)	✓	✓
Double exponential (BL)	✓	✗
Gaussian mixture (BayesC)	✓	✓
Scaled-*t* mixture (BayesB)	✓	✓
Stochastic Search Variable Selection (SSVS)	✗	✓
Reproducing Kernel Hilbert Spaces (RKHS)	✓	✓

The BGLR package was originally developed for regression problems involving many more predictors (*p*) than sample size (*n*, i.e. p>n). Therefore, the BGLR function simulates posterior samples using computations on individual genotype–phenotype data. Many genomic prediction tasks, including the development of polygenic scores (PGS) with genome-wide association studies (GWAS)-selected SNPs and biobank-size data as well as many large-scale animal and plant genetic evaluations, present problems where sample size exceeds the number of predictors (n>p). In such settings, the computational performance and the speed of algorithms can be improved by simulating posterior samples from sufficient statistics (SS) ($y^{\prime }y$, $X^{\prime }X$, and $X^{\prime }y$). This approach is not new (e.g. Vilhjámsson *et al.* 2015); however, it has not been available within BGLR. Additionally, software that uses SS as inputs can enable the joint analysis of multiple data sets without the need to share individual genotype–phenotype data which can pose privacy and security concerns. Therefore, to offer efficient software for biobank-sized data and to enable analysis without sharing individual data, we developed the BLRXy function that has a user interface identical to that of BGLR but, unlike BGLR, it simulates posterior samples from SS.

## Models and methods

The BLRXy function fits single-trait linear regression models of the form:


(1) y=Xβ+ε,

where $y=(y_{1},\ldots ,y_{n}{)}^{\prime }$ is an *n*-dimensional vector of phenotypes, $X=\{x_{ij}\}$ is a matrix of predictors of dimension n×p, $\beta =(\beta_{1},\ldots ,\beta_{p}{)}^{\prime }$ is a *p*-dimensional vector of effects, and $\varepsilon =(\varepsilon_{1},\ldots ,\varepsilon_{n}{)}^{\prime }$ is an *n*-dimensional vector of error terms.

For a quantitative (possibly transformed) trait, we assume that the errors are IID (identically and independently distributed) normal $\varepsilon_{i}∼N(0,\sigma_{\varepsilon }^{2})$; therefore, the conditional distribution of the data given the model parameters $\theta =\{\beta ,\sigma_{\varepsilon }^{2}\}$ is:


(2) $$\begin{array}{rl} \;p(y\;|\;\theta ) & =MVN(y\;|\;X\beta ,I\sigma_{\varepsilon }^{2})\\ & =(2\pi \sigma_{\varepsilon }^{2}{)}^{-n/2}\exp\;\left\{-\frac{1}{2\sigma_{\varepsilon }^{2}}(y-X\beta {)}^{\prime }(y-X\beta )\right\}\\ & =(2\pi \sigma_{\varepsilon }^{2}{)}^{-n/2}\exp\;\left\{-\frac{1}{2\sigma_{\varepsilon }^{2}}(y^{\prime }y+\beta^{\prime }X^{\prime }X\beta -2X^{\prime }y)\right\}. \end{array}$$

In the above model, the likelihood function depends on the data only through the SS $y^{\prime }y$, $X^{\prime }X$, and $X^{\prime }y$; therefore, all the computations needed to simulate samples from the posterior distribution can be performed using SS.

### Prior distributions and posterior inferences

The BGLR R-package implements seven different prior distributions of effects (Table 1). The priors that are implemented in the BLRXy function, the rules used to set default values to hyper-parameters are the same as those implemented in BGLR; therefore, we refer to Pérez and de los Campos (2014) for further details about the priors implemented. Likewise, the class of algorithms used to generate posterior samples in BLRXy are the same as those used in BGLR, primarily Gibbs sampling (Geman and Geman 1984) and, for some parameters in some models, Metropolis–Hastings (Chib and Greenberg 1995). However, the BLRXy function performs all the computations needed to simulate posterior using the SS. Further details about the computations needed to simulate posterior samples are presented in Supplementary Section 1.1 of the Supplementary Methods.

## Examples

The example in Box 1 shows how to fit a linear regression on SNPs using a Gaussian prior.

Box 1. **Fitting regression model with Gaussian prior with BLRXy**

library(BGLR)

data(wheat)

X<-scale(wheat.X,center=TRUE,scale=FALSE)

y<-wheat.Y[,1]

# As in BGLR, BRR is the keyword for Gaussian prior

LP<-list(list(X=X,model=“BRR”))

fm1<-BLRXy(y=y,ETA=LP)

In the above example, the model was fitted using the BLRXy function; however, the same model can be fitted using BGLR as shown in Box 2.

Box 2. **Fitting regression model with Gaussian prior with BGLR**

fm2<-BGLR(y=y,ETA=LP)

There is an important difference though, when the model is fitted using BLRXy, this function derives the SS ($y^{\prime }y$, $X^{\prime }X$, and $X^{\prime }y$) and makes a call to the function BLRCross which generates posterior samples using SS as inputs.

### Fitting models using SS as inputs

The function BLRXy provides a convenient interface; however, for some applications, a direct call to the function BLRCross is useful (we will present examples later in this article). The script in Box 3 shows how to fit a model equivalent to fm1 (of Box 1) using the BLRCross function.

Box 3. **Fitting regression model with Gaussian prior with BLRCross**

p<-ncol(X)

XX<-crossprod(X)

Xy<-crossprod(X,y)

idPriors<-rep(1,p)

priors<-list(list(model=“BayesC”))

fm3<-BLRCross(my=mean(y),vy=var(y),n=length(y),

XX=XX,Xy=Xy,idPriors = idPriors,priors = priors)

Above:

- p is the total number of predictors included in the model,
- idPriors is a *p*-dimensional vector of integers used to identify set of predictors assigned different priors, and
- priors is a list of priors, with one entry in the list corresponding to the index idPriors. Specifically, the prior used for all the predictors having a value *s* in idPriors is specified in the *s*th entry of the priors list.

It is also possible to provide directly a vector of phenotypes (y) to the function. In that case, the user is not required to provide the sample mean (my), the variance (vy), and the sample size (n) as these can be derived from the phenotype vector.

### Fitting models with multiple random effects

Similar to BGLR, the BLRXy and BLRCross allow users to specify different priors for subsets of the predictors. The code in Box 4 shows how to fit models with three sets of predictors. To illustrate, we split the SNPs into three sets to which we assign different priors.

Box 4. **Fitting linear models with different priors with BLRXy**

X1<-X[,1:5]

X2<-X[,6:100]

X3<-X[,101:500]

LP<-list(list(X=X1,model=“FIXED”),

list(X=X2,model=“BayesC”),

list(X=X3,model=“BRR”))

fm4<-BLRXy(y=y,ETA=LP,verbose=FALSE)

Users can also fit the above model using the BLRCross function directly; this is illustrated in Box 5.

Box 5. **Fitting linear models with different priors with BLRCross**

idPriors<-c(rep(1,ncol(X1)), rep(2,ncol(X2)),

rep(3,ncol(X3)))

priors<-list(list(model=“FIXED”),

list(model=“BayesC”),

list(model=“BRR”))

W<-cbind(X1,X2,X3)

XX<-crossprod(W)

Xy<-crossprod(W,y)

fm5<-BLRCross(my=mean(y),vy=var(y),n=length(y),

Xy=Xy,XX=XX,

idPriors=idPriors,priors=priors)

## Benchmarks

We first present a benchmark of BLRXy, a sampler that simulates posterior samples using SS as inputs, against BGLR which simulate posterior samples using individual genotype–phenotype. Then, we present a benchmark of BLRXy against LDPred—one of the most popular software packages for developing PGSs (Privé *et al.* 2020).

### Benchmark 1: posterior sampling using individual genotype–phenotype data vs using SS

We compared the computational performance of the sampler implemented in the BLRXy function against the one implemented in BGLR over different scenarios regarding sample size and the number of predictors. These benchmarks were conducted in Michigan State University’s high-performance computing cluster using nodes equipped with Intel Xeon Gold 6148 processors at 2.40 GHz and 128 GB of RAM. All the analyses presented in this study were done using R-4.4.1 (R Core Team 2024), linked against the OpenBLAS library (https://www.openblas.net).

The data used to benchmark the software consisted of real genotypes from human chromosome 1 from the UK-Biobank (Sudlow *et al.* 2015). Using these genotypes, we simulated a trait with 50 causal loci and a trait heritability of 5%. We simulated data and fitted models for 16 scenarios, obtained by combining four values for the number of SNPs (p=1K,3K,5K,10K, K=1,000) and four sample sizes (n=10K,50K,100K,300K).

For each of these scenarios, we ran 30 Monte Carlo (MC) replicates and recorded the time it took to derive SS and the time required to generate 3,000 samples using either BLRXy or BGLR using a single thread. For both samplers, we used variable selection prior BayesC (Table 1). The scripts used to conduct these benchmarks are provided in Supplementary Section 4.2 of the Supplementary Materials.

**Fig. 1.**
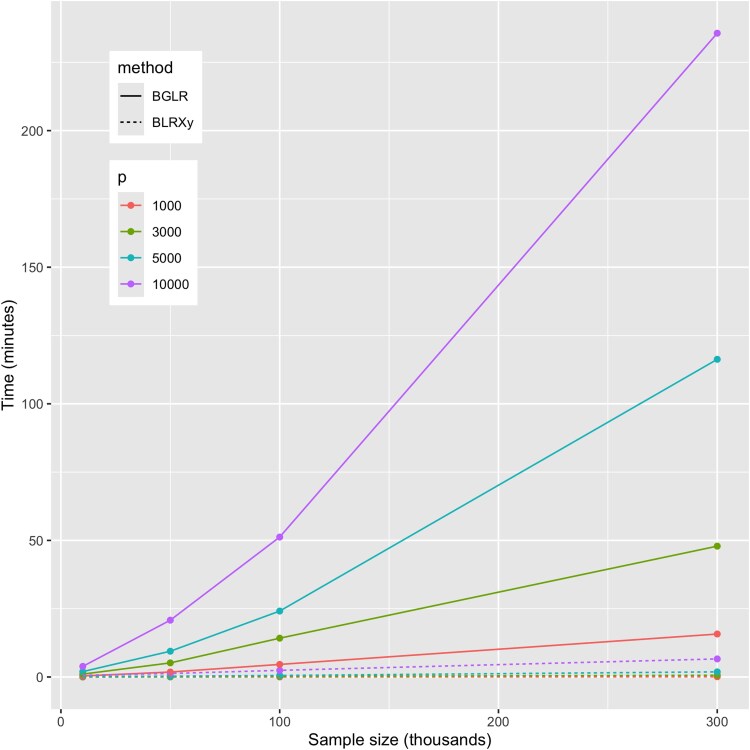
Computing time required to simulate 3,000 posterior samples using the BLRXy and BGLR functions when using one thread (see Supplementary Fig. S1 relative times). BLRXy derives SS and then generates posterior samples using SS as data. BGLR simulates posterior samples using individual genotype–phenotype data as inputs.

Across scenarios, the computing time used by BLRXy was substantially smaller than the one used by BGLR, even for cases where the number of markers was the same as the sample size (e.g. p=n=10K, Fig. 1, see also Supplementary Table S1 and Fig. S1). In scenarios where p<n, BLRXy was much faster, up to 150 times faster for the scenario involving 1K SNPs and a sample size of 300K (Supplementary Fig. S1).

**Fig. 2.**
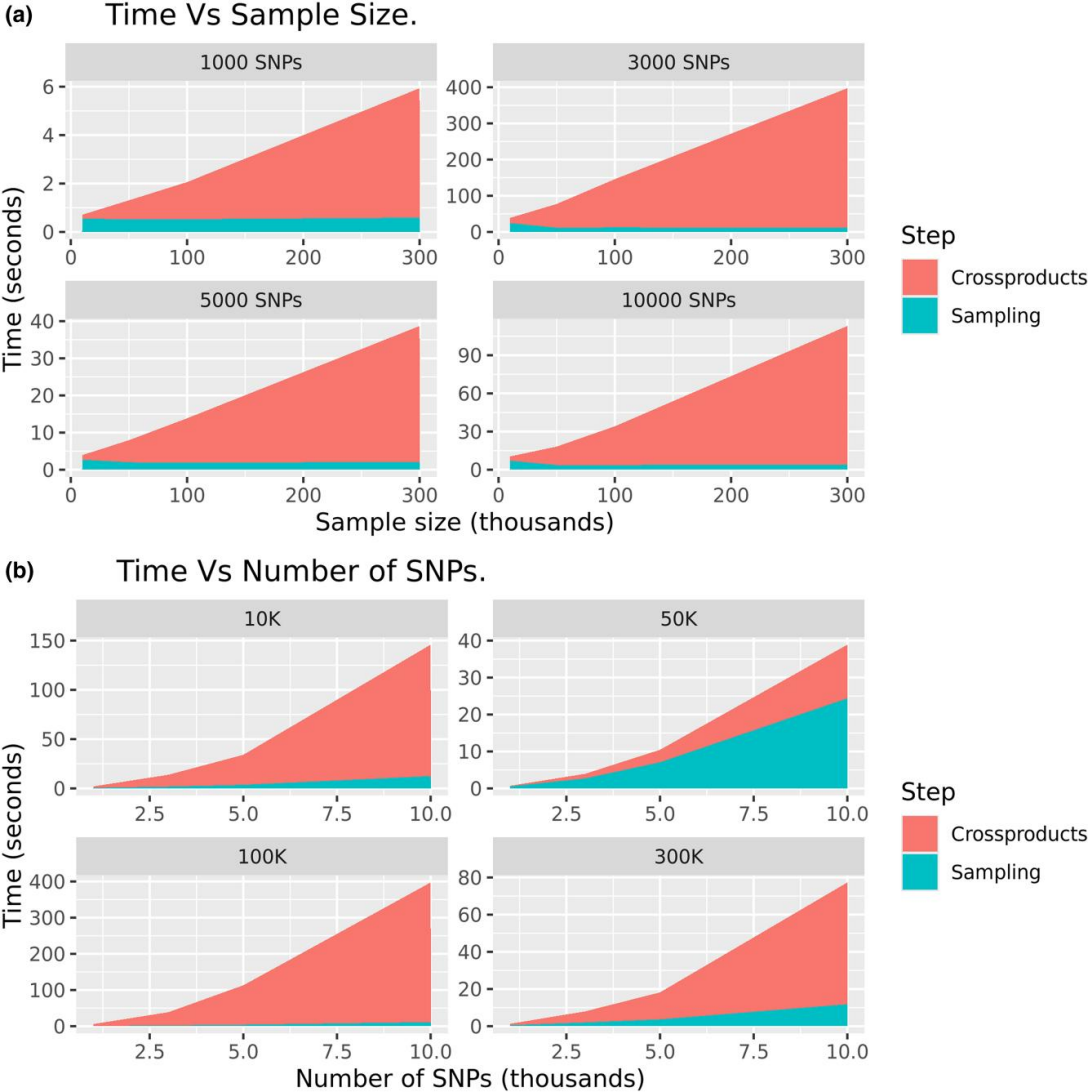
Computing time (in seconds) required to derive SS and to generate 3,000 posterior samples using the BLRCross function. The prior used was BayesC and the computations were done using a single thread. Panel (a) shows time vs sample size and panel (b) shows time vs number of SNPs.

In Fig. 2, we decomposed the total time used by BLRXy to simulate 3,000 posterior samples into the time it took to derive the SS and the time used to simulate the samples from the SS. As one would expect, the time required to simulate posterior samples using the BLRCross function depends on the number of SNPs and not on sample size (compare the blue areas in Fig. 2a and b). On the other hand, the time required to compute the SS depends on both *n* and *p*, this was expected because the computation of $X^{\prime }X$ has a $O(np^{2})$ complexity.

In most of the scenarios that we evaluated, the time it took to derive SS was much higher than the time it took to simulate posterior samples (Fig. 2). For the benchmarks presented in this section, we generated 3,000 posterior samples per replicate. In practical applications, a larger number of samples is usually collected; in these cases, the advantage of using BLRXy relative to BGLR will be much larger than what is shown in Fig. 1 and Supplementary Fig. S1 because BGLR computing time is proportional to the number of iterations. This is not the case for BLRXy because the time used to derive SS (which is often the most demanding step) does not depend on the number of posterior samples simulated. For instance, using the results in Supplementary Table S1, it took 6.4 min for BLRXy to derive SS (using one thread) and 34 s to simulate 3,000 posterior samples making a total of 6.6 min. Since the sampling time is directly proportional to the number of iterations, it will take ∼16 min (6.4 min to derive SS and 5.6 min for sampling) to simulate 50,000 samples using BLRXy on a single thread.

The results in Figs. 1 and 2 were obtained using a single thread. We repeated the benchmark, setting the number of threads used by OpenBLAS equal to four. Using four threads produces an important reduction in the time required to compute the SS. Thus, using four threads BLRXy was more than 300 times faster than BGLR when the number of SNPs was 1,000 and the sample size was 300,000 (Supplementary Fig. S2). For the most demanding scenario (n=300,000 and p=10,000) it took 6.6 min for BLRXy to derive SS and to simulate 3,000 posterior samples when using one thread (Supplementary Table S1); the same task took 3 min when four threads were used (Supplementary Table S2).

We repeated the benchmark presented above using a Gaussian prior which, unlike the BayesC prior used in the benchmark presented above, does not perform variable selection. The computing times were larger for both BGLR and BLRXy when using a Gaussian prior (Supplementary Table S3), compared with those observed when using a variable selection prior (Supplementary Table S1). This was expected because with variable selection priors only a fraction of the SNPs are concurrently active, thus reducing the computational burden of the sampling process. However, the advantage of using BLRXy over BGLR when p>n was still very clear.

### Benchmark 2: BLRCross versus LDPred-auto

LDPred (Privé *et al.* 2020) is a software commonly used to develop PGS using summary (or sufficient) statistics as inputs. For Bayesian PGSs, LDPred uses a variable selection prior of the spike–slab family similar to the BayesC prior implemented in BGLR R-package. Therefore, we benchmark the sampler we implemented in the BLRCross function of the BGLR package against LDPred-auto both in terms of prediction accuracy and the computational time required to simulate posterior samples. We did not include in the benchmark for computational times the time it takes to derive the SS because these are done with other functions and computational complexity and times are equivalent for the two packages.

The training data set used in this benchmark consisted of ∼330,000 individuals of European ancestry from the UK-Biobank, and the testing set consisted of 10,000 individuals from the same cohort and ancestry group that were distantly related (GRM<0.05) among them and with those of the training set. We benchmarked the two software using eight traits: Body Mass Index (BMI), Cholesterol, Creatinine, HDL-cholesterol (HDL), Standing Height (Height), LDL-cholesterol (LDL), Systolic Blood Pressure (SBP), and Triglycerides. These phenotypes were preadjusted by sex, age, recruitment center, and 10 SNP-derived PCs. For each of these phenotypes, we built a PGS using the SNPs that had a single-marker-regression association $P\text{-value}<1\times 10^{-5}$ (de los Campos *et al.* 2023). Subsequently, we estimated the effects of the selected SNPs using LDPred-auto and BLRCross using data from the training set and evaluated the prediction performance in the testing data set (n=10,000 distantly related individuals of European ancestry) whose data was not used in SNP selection of effect estimation.

LDPred can use sparse LD matrices where small (absolute-value) correlations between SNPs may be zeroed out to improve computational efficiency. However, ignoring LD between distant markers may compromise prediction performance and can make algorithms unstable. We considered two strategies for LDPred: *dense*, which fully accounts for LD among all the SNPs included in the PGS, and *chromosome block-diagonal*, for which we zeroed-out LD among SNPs from different chromosomes. We also tried a *radius* approach (Vilhjámsson *et al.* 2015) which uses a band-diagonal matrix accounting for LD with 100 flanking SNPs around each of the variants included in the PGS; however, with this strategy, LDPred was unstable, leading to NAs for estimated coefficients for all the traits we analyzed; therefore, we only present results for *dense* and *chromosome block-diagonal* strategies. For BLRCross, we just considered dense SS because, in the current implementation, our software does not allow for sparse SS.

**Fig. 3.**
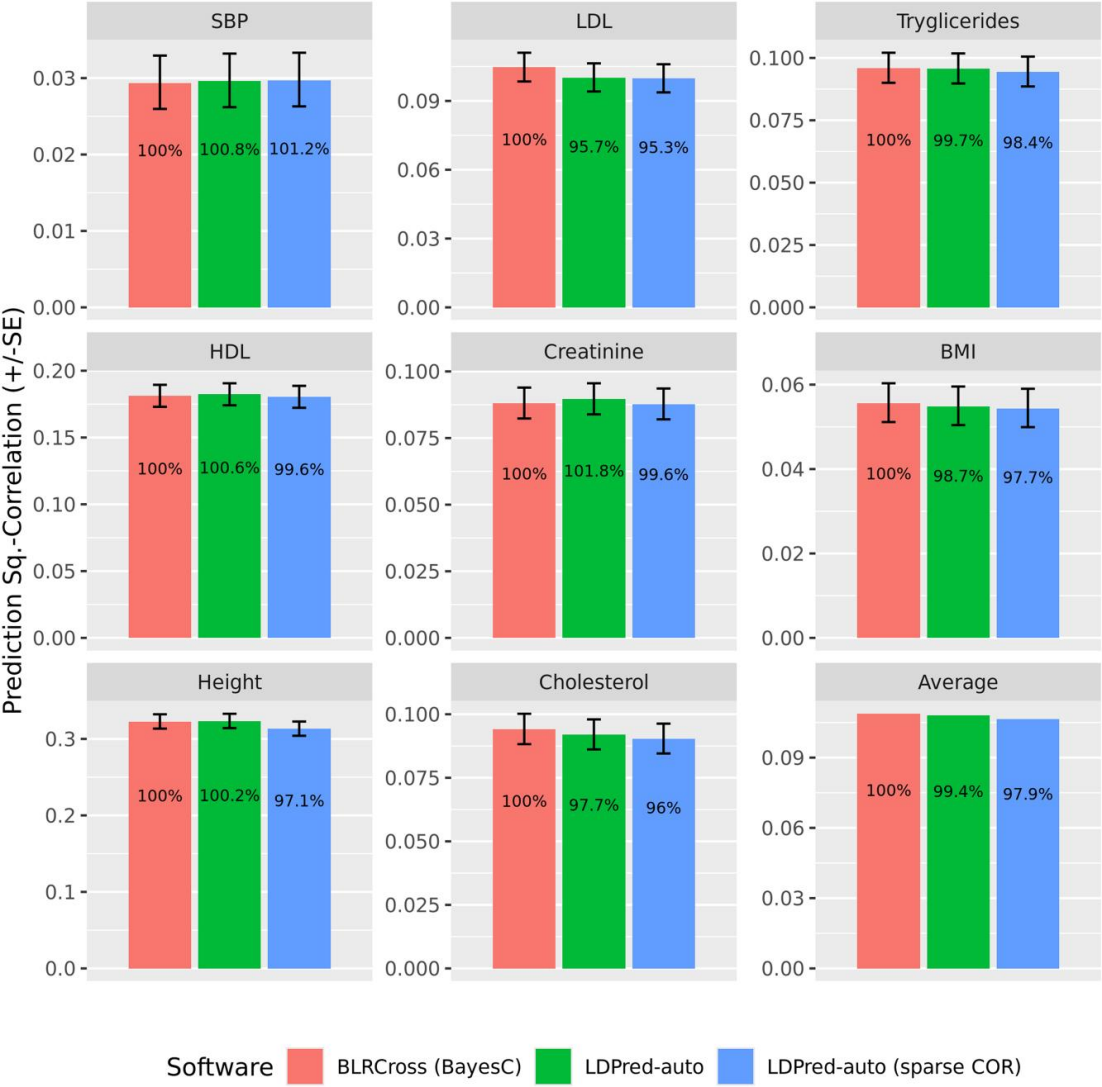
Prediction $R^{2}$ (±SE) of PGS derived using LDPred-auto and BLRCross by trait. The numbers within the bar are the $R^{2}$ values presented as % of the $R^{2}$ achieved by BLRCross (BayesC prior).

Figure 3 shows the prediction correlations we obtained by trait and software. There were no sizable differences between the prediction accuracies achieved using BLRCross and LDPred-auto; this was not surprising given that both software use the same likelihood and, for this benchmark, for BLRCross we used the BayesC prior which is very similar to the one used by LDPred-auto. The use of sparse LD matrices resulted in small reductions in prediction accuracy—the average reduction in prediction $R^{2}$ was 2.1%, compared with BLRCross, see the lower right panel in Fig. 3.

For most traits, individual PGS ranged approximately one SD around the mean; however, the spread of PGSs was larger for height and smaller for SBP (Supplementary Fig. S3), which is expected given the higher (lower) heritability of height (SBP).

Recent studies (Ding *et al.* 2022) highlighted the importance of quantifying uncertainty about individual PGS-Bayesian models are particularly adequate for this task. To illustrate this, we run 10 chains of the BayesC model whose prediction results are depicted in Fig. 3, and used the posterior samples of SNP effects to estimate 95% credibility regions of individual PGS for each of the traits (Supplementary Fig. S3). As noted by Ding *et al.* (2022), a fraction of the PGS in the top 10% has a lower-bound of the 95% CI above the top-10th percentile of the PGS.

We also benchmarked BLRCross against LDPred-auto in terms of computational time. For this benchmark, instead of using all the SNPs with GWAS $P\text{-value}<1\times 10^{-5}$, for each of the eight traits, we ranked SNPs based on GWAS *P*-values (from smallest to the largest) and fitted models using the top-*k* SNPs of the rank (k=1,000, 2,000, 3,000, 5,000, 10,000). For each trait, SNP set, and software, we recorded the time it took to simulate 25,000 posterior samples. To account for variability in the computational time due to the computing node used to perform the benchmark, we ran 30 replicates for each SNP set and trait.

**Fig. 4.**
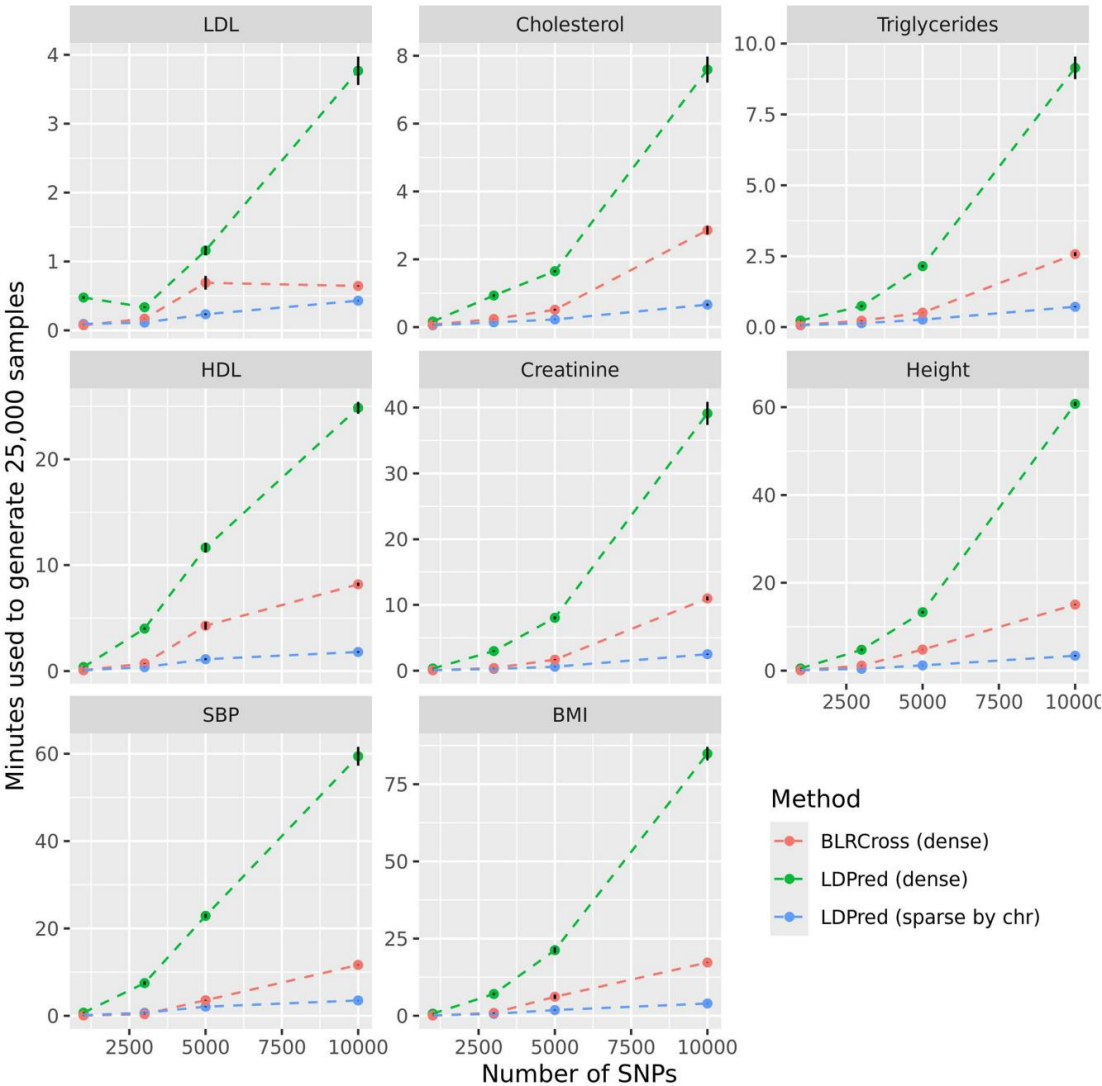
Computational time (±SD) needed to simulate 25,000 posterior samples by trait, software, and number of SNPs. Both methods used a variable selection prior with a Gaussian slab, and computations were done using a single thread.

Figure 4 shows the computational time used by each software by trait and number of SNPs. In all cases, BLRCross was considerably faster than LDPred-auto*dense* but slower than LDPred-auto*sparse*. In general, the algorithm implemented in BLRCross was 2–4 times faster than the one implemented in LDPred-auto when using dense SS. There were important differences in the computational time used by each software between traits. This was primarily due to the trait architecture and the corresponding model sparsity. For instance, with BLRCross and for the model using 10,000 SNPs, the (estimated) posterior mean of the percentage of SNPs with nonzero effects was 24.4, 28.9, 15.6, and 15.5%, for BMI, Height, SBP, and Creatinine, respectively, and only 2.5, 3.1, 3.6, and 4.7%, for LDL, Cholesterol, HDL, and Triglycerides, respectively.

## Polygenic scores using hundreds of thousands of SNPs

In the previous section, we presented results for PGS using SNPs with GWAS association $P\text{-value}<1\times 10^{-5}$. For highly complex traits such as height or BMI there can be further gains in prediction accuracy by including SNPs with larger GWAS association *P*-values (e.g. $P\text{-value}<1\times 10^{-2}$) or even using all the available SNPs. However, this may require fitting hundreds of thousands of SNPs jointly which presents both statistical and computational challenges.

As noted in the previous section, a common approach used to reduce the computational burden involved when estimating SNP effects is to use sparse LD matrices which ignore LD between distant SNPs. For instance, for small-effects variants, DBSLMM assumes that $X^{\prime }X$ is block-diagonal which enables parallelization. Another approach is to fit models to overlapping chromosome segments consisting of a core and flanking regions (Funkhouser *et al.* 2020; de los Campos *et al.* 2023), see Supplementary Fig. S4.

From a modeling perspective, using hundreds of thousands of SNPs may lead to excessive shrinkage of large-effect variants when a Gaussian prior distribution is used. To avoid this problem, one can use thick-tailed priors or finite mixture, variable selection priors (Table 1). The samplers implemented in the BGLR R-package offer the possibility of assigning different priors (and estimating separate hyper-parameter values) for user-defined SNP sets (see examples in Boxes 4 and 5). This feature can be used to prevent excessive shrinkage of large-effect variants. For example, Kim *et al.* (2017) and Yang and Zhou (2020) used GWAS summary statistics to classify SNPs into putative large- and small-effect variant sets and assigned set-specific priors (and hyper-parameters)—we followed these strategies in the following example.

To illustrate the impact using hundreds of thousands of variants, we fitted PGS including all the array variants after QC (784256 SNPs). The genotype and phenotype data (as well as the training and testing sets) used for the analysis presented in this section are the same as the ones for our Benchmark 2 of the previous section.

Following Funkhouser *et al.* (2020) and de los Campos *et al.* (2023), we estimated SNP effects by fitting models to overlapping chromosome segments of 5,000 variants (3,000 core variants, ∼11.5 Mbp, plus 1,000 variants, ∼3.8 Mbp, on each of the flanking regions, see Supplementary Fig. S4). For each segment, we saved estimates from the core and discarded estimates from the flanking regions (see Supplementary Section 1.2 of the Supplementary Methods for more details). We considered four approaches:

- *No sets*: For this method, we estimated SNP effects for each of the cores (using the overlapping segments approach described above) without any distinction into putative large- and small-effect SNPs. We also considered three methods that grouped SNPs into putative large- and small-effect size sets.
- *All SNPs (w/clumping)*: For this method, following Yang and Zhou (2020), we identified the large-effect SNPs using *P*-value thresholding $\left(P\text{-value}<5\times 10^{-8}\right)$ and LD clumping (removing all SNPs with an $R^{2}$ with leading variants >0.1), and fitted models that included the large-effect SNPs and all the SNPs within one chromosome segment that were not part of the large-effect SNPs set. To avoid excessive shrinkage of the large-effect variants, we used set-specific hyper-parameters using the BayesB prior (see Supplementary Section 1.2 of the Supplementary Methods for further details).
- *All SNPs (no LD clumping)*: This method is similar to *All SNPs (w/clumping)* but we did not perform clumping. Thus, SNP-set formation was simply based on *P*-value thresholding as done in Kim *et al.* (2017). Compared with *All SNPs (w/clumping)*, this approach is simpler to implement (it does not require the clumping step) and may be better at capturing signals at large-effect loci; however, it is computationally more demanding.
- *All-SNP (2-steps PGS)*: For this 2-step method, we first derived a PGS for the large-effect SNP set (obtained through *P*-value thresholding plus LD clumping as in *All SNPs w/LD clumping)*. Then, in the second step, we estimated the effects of all the other SNPs by fitting models to overlapping chunks of 5,000 variants and the fixed effect of the PGS computed in the first step. This method is computationally much faster than any of the other two methods.

For the methods described above, we used the BayesB prior which is a variable selection prior with a thick-tailed (a scaled-*t* distribution with 5 df) slab. For models involving two sets, separate hyper-parameters (both the proportion of SNPs with nonzero effects and the scale parameter of the *t*-distribution) were estimated for the large- and small-effect SNP sets.

###  

#### Results

**Fig. 5.**
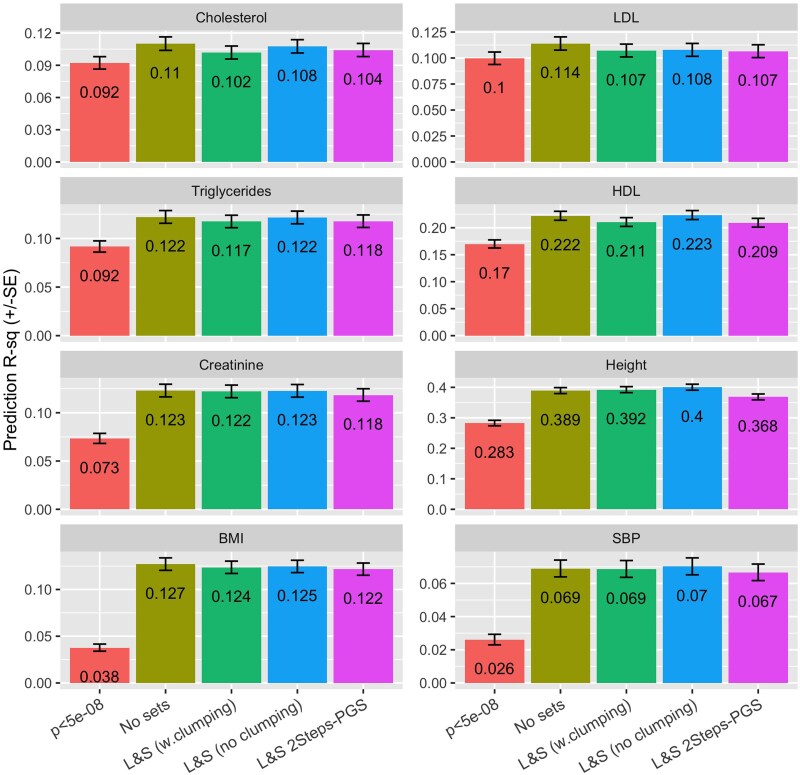
Prediction $R^{2}$ (±SE) by trait and method. Method: $P\text{-value}<5\times 10^{-8}$ is a PGS with all GWAS-significant SNPs fitted jointly, the other four methods use all the SNPs from the UK-Biobank array (after QC) and were fitted using overlapping chromosome segments of 5,000 SNPs (3,000 in the core, and 1,000 in each of the flanking regions). The prior used was BayesB with different hyper-parameters estimated for the (putative) small- and large-effect SNP sets.

Compared with PGS using only GWAS-significant SNPs, using all genome-wide SNPs led to gains in PGS prediction $R^{2}$ which were moderately high for cholesterols, and much larger for creatinine, height, BMI, and SBP (Fig. 5). The differences in the prediction performances of the four methods that used all the available array variants were moderately small. The overall best methods were *No sets* and *All SNPs (no LD clumping)*, and the method with overall worst prediction performance was *All-SNP (2-steps PGS)*. However, as noted, the differences between the four methods were small. In general, it appears that LD clumping of the large-effect SNPs resulted in a small reduction in prediction $R^{2}$.

The prediction $R^{2}$ values that we achieved are comparable with those reported by recent studies that used big data to derive PGS for the traits that we analyzed. For instance, for height, we obtained an $R^{2}$ close to 0.4, which is higher than the $R^{2}$ reported for the same trait and data set by Yang and Zhou (2020) ($R^{2}$ = 0.302) and smaller than the one reported by Yengo *et al.* (2022) who, using data from 5.4 million individuals, reported a PGS $R^{2}$ for height of 0.45. Likewise, the $R^{2}$ we reported for BMI (∼0.125) was higher than the one reported by Yang and Zhou (2020) (0.108) for the same trait and data set.

The average time it took to simulate 25,000 posterior samples for chromosome segment varied from 3 min (for LDL) to 30 min for Height (*Sets no LD clumping*), see Supplementary Fig. S5. There were important differences in computing time both between traits (with traits for which the fitted models were sparser taking considerably less times than those for which the estimated proportion of nonzero effects was larger) as well methods. Overall the *No sets* methods were the fastest method, taking between 3 and 6 min to generate 25,000 posterior samples, depending on the trait. This happened because in this method only the SNPs in the chromosome segment (5,000) were included in the model, while, for *All SNPs (w/clumping)* and *All SNPs (no LD clumping)*, each run included the 5,000 SNPs of the chromosome segment being fitted plus all the SNPs in the large-effect SNPs (see Supplementary Table S4 for the number of SNPs in the large- and small-effect SNPs by trait and method).

## Joint analysis of multiple data sets using SS

Sample size is a major factor affecting the accuracy of effect estimates and prediction accuracy. One way to achieve a larger sample size (and possibly a more diverse training data set) is by analyzing data from multiple cohorts jointly. However, sharing individual genotype–phenotype data is not always possible. In this section, we show how the BLRCross function can be used to analyze data from several data sets jointly without sharing individual genotype–phenotype data.

To illustrate, consider two data sets $D_{1}=\{X_{1},y_{1}\}$ and $D_{2}=\{X_{2},y_{2}\}$ and assume that the columns of the two genotype matrices ($X_{j},j=1,2$) are matched and that the phenotype vectors are on the same scale and have been preadjusted by systematic effects (e.g. sex, age). Suppose we wish to fit a linear model (1) using data from both data sets, with $y^{\prime }=(y_{1}^{\prime },y_{2}^{\prime })$ and $X^{\prime }=(X_{1}^{\prime },X_{2}^{\prime })$. The SS for this problem are $y^{\prime }y=y_{1}^{\prime }y_{1}+y_{2}^{\prime }y_{2}$, $X^{\prime }X=X_{1}^{\prime }X_{1}+X_{2}^{\prime }X_{2}$, and $X^{\prime }y=X_{1}^{\prime }y_{1}+X_{2}^{\prime }y_{2}$.

Therefore, we can analyze the two data sets jointly by first deriving the SS for each data set, and then fitting the model by adding data set-specific SS. A simple demonstration of how SS from multiple sources can be combined and used to fit models using the BLRCross function is presented in Supplementary Section 4.1 of the Supplementary Materials and a large-scale application is presented in the following section.

### Improving polygenic score prediction for Hispanics leveraging large data from European ancestry

In this section, we present a large-data example involving the joint analysis of data from the UK-Biobank (Sudlow *et al.* 2015), All of Us (AOU, The All of Us Research Program Investigators 2019), and the Hispanic Community Health Study/Study of Latinos (HCHS/SOL, Gallo *et al.* 2019). Hispanics are severely under-represented in GWAS. The example shows how integrating data from multiple data sets can improve PGS prediction accuracy for a group that is under-represented in genomic study sets.

#### Data and methods

We derived PGS for the eight traits previously analyzed using data of Hispanics subjects from the HCHS/SOL cohort (n∼12,000), Hispanics from the AOU cohort (*n* ranged from ∼11,000 to ∼57,000 depending on the trait, Supplementary Table S5), and distantly related Europeans from the UK-Biobank.

All the traits were preadjusted by sex and age within cohort. We identified ∼650,000 SNPs that were present in the array used in AOU and the imputed genotypes from the HCHS/SOL cohort and the imputed genotypes from the UK-Biobank.

To evaluate prediction accuracy, we randomly assigned the data from HCHS/SOL to five folds using a stratified sampling approach that maintains the representation of each of the regions of origin (Central America, Cuban, Dominican, Mexican, Puerto Rican, South America, and other origin). To derive the PGSs, we:

- Performed Genome-Wide Association (GWA) analysis for each of the traits using data from UK-Biobank and included in the PGS all the SNPs with a $P\text{-value}<1\times 10^{-5}$.
- Derived the SS for the AOU (Hispanics), the UK-Biobank (Europeans), and for each fold of the HCHS/SOL cohort.
- Estimated SNP effects using BLRCross with a prior from the Spike–Slab family (model BayesC, see Habier *et al.* 2011). For each trait, we derived seven PGSs: three PGS derived using SS from only one of the data sets, three combining SS from two data sets, and one PGS derived by combining the SS from the three data sources.
- Finally, we evaluated the prediction accuracy of the resulting PGS in each of the left-out folds by correlating the predictions with the adjusted phenotype within the region of origin. From these analyses, we report the (weighted, with weights proportional to sample size) average of the squared correlations.

#### Results

**Fig. 6.**
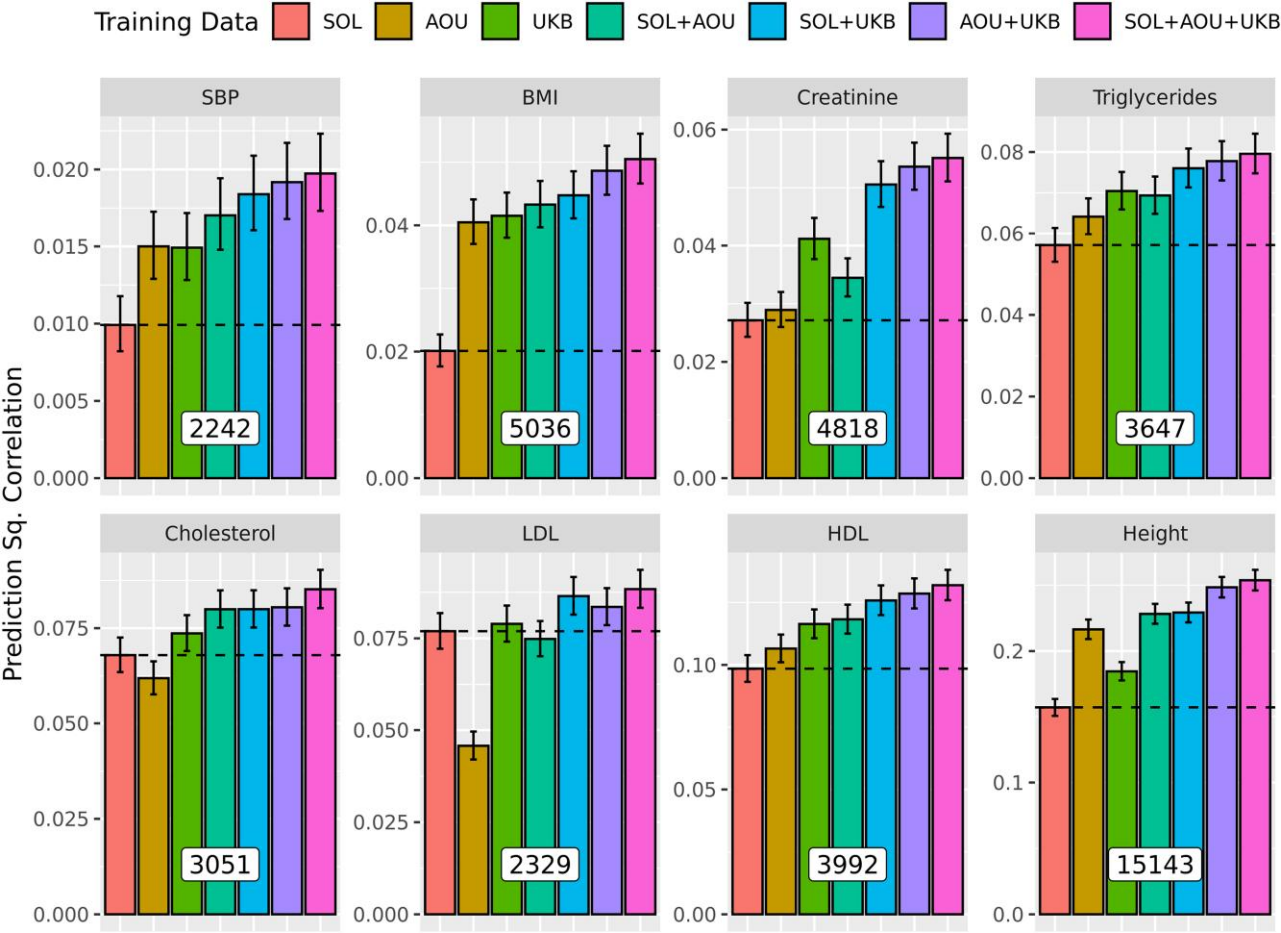
Prediction $R^{2}$ (±SE) of Polygenic Scores in testing data from the HCHS/SOL cohort by trait and training strategy. The number of SNPs used (i.e. those with $P\text{-value}<1\times 10^{-5}$), are provided at the base of the bars in the white boxes. The prior used was BayesC.

The number of SNPs that had $P\text{-value}<1\times 10^{-5}$ ranged from ∼2,242 (SBP) to ∼15,143 (Height, Fig. 6). For all traits, the prediction $R^{2}$ achieved by combining data from two or three sources were higher than the one achieved using data from the HCHS/SOL cohort only (Fig. 6). Combining the SS from the three cohorts, led to significant (and substantial) gains in prediction accuracy ranging from 14.9% (LDL) to 151.0% (BMI), both relative to the PGS prediction $R^{2}$ achieved for the same trait using HCHS/SOL data only (Supplementary Table S6).

## Discussion

We present a new implementation within the BGLR R-package that is tailored for problems involving sample sizes larger than the number of predictors used in the model. The implementation performs all the computations needed to simulate posterior samples using SS. The computational speed that can be achieved with this strategy can be orders of magnitude faster than that of algorithms that perform computations on individual genotype–phenotype data. Thus, for problems involving n>p, we recommend using BLRXy (or BLRCross) instead of the BGLR R-function. Having the option to perform computations using SS also enables multiple analyses that cannot be implemented using software that requires individual genotype–phenotype data; next, we discuss a few examples.

*Joint analysis of multicohort data without sharing individual genotype–phenotype data*: Since the SS required to fit linear models are sums over subjects, SS can be shared without compromising privacy. Therefore, the software that we present in this study can be used to analyze multiple data sets jointly even in cases where sharing individual data poses privacy concerns. It is important to highlight that analysis based on SS produces results that are equivalent to the ones obtained by analyzing all the individual genotype–phenotype data jointly.

The joint analysis that we presented implicitly assumed that SNP effects are homogeneous across data sets. This assumption may not be optimal for ancestry-diverse data. To contemplate effect heterogeneity one can use multitrait models (e.g. de los Campos, Veturi, *et al.* 2015; Runcie *et al.* 2021) or interaction models (e.g. de los Campos, Veturi, *et al.* 2015; Veturi *et al.* 2019). The BGLR R-package offers the Multitrait function which can be used for such analyses using individual genotype–phenotype data; however, multitrait models are computationally demanding and difficult to implement using SS. Another approach is to weighted SS, with weights assigned depending on the ancestry of the individuals used to derive the SS (more below) or using Transfer Learning techniques such as the ones proposed in Zhao *et al.* (2022).

*Deriving PGS from GWAS summary statistics and reference panels*: One of the SS required to fit linear models ($X^{\prime }y$) can be derived from GWAS results and the other SS ($X^{\prime }X$) can be approximated using reference panes (e.g. Vilhjámsson *et al.* 2015). Such approximate analysis can be performed using the software presented in this study. However, it is worth noting that when there are systematic differences between the samples from the reference panels and those used in the GWA study and the number of SNPs is large, the approximation to $X^{\prime }X$ can be noisy and the results obtained can be sub-optimal.

*Sequential analysis*: In genetic evaluations in plants and animals, in every generation new individuals are genotyped, and either those individuals or their relatives (e.g. progeny) are phenotyped. Sequential analysis of such data can be streamed lined by updating SS (by adding to the previous SS the contribution of the new data that is produced in each generation) and then fitting models using the updated SS. For genetic evaluations using data from hundreds of thousands of individuals and a few thousand SNPs (a common situation in many plant and animal breeding programs), this strategy could enable very fast computations.

*Weighted analysis*: Above, and in the example combining data from the UK-Biobank, AOU, and the HCHS/SOL cohort, we assume that the observations from different sources of information have equal information value. However, in some contexts, it may be better to weigh different sources of information differently. For instance, in the combined analysis of UK-Biobank and the HCHS/SOL data, since the target population is Hispanics, one may assign higher weights to data from the HCHS/SOL and AOU-Hispanic than to data from the UK-Biobank. Likewise, in sequential analysis, one may wish to down-weight SS coming from older generations.

*Transfer Learning*: The possibility of modeling multiple random effects jointly (including random effects with flat priors) can be leveraged to transfer learning between data sets and populations. For instance, imagine that we have access to two previously developed PGS (represented by ${\hat{\beta }}_{1}$ and ${\hat{\beta }}_{2}$) and we want to develop a new PGS using a different data set {X,y} leveraging the information provided by the two existing PGSs. To achieve this, one could fit a model of the form $y=1\mu +{PGS}_{1}\alpha_{1}+{PGS}_{2}\alpha_{2}+Xb+\varepsilon$, where ${PGS}_{j}=X_{j}{\hat{\beta }}_{j}$ (j=1,2), with $\alpha_{1}$ and $\alpha_{2}$ treated as “fixed” effects and assigning b a shrinkage (or variable selection+shrinkage) prior. The resulting estimated effects of the new PGS will be defined as $\hat{\beta }=\hat{\alpha_{1}}{\hat{\beta }}_{1}+\hat{\alpha_{2}}{\hat{\beta }}_{2}+\hat{b}$. We have evaluated this approach to derive PGS for Hispanics using UK-Biobank derived PGS as fixed effects and it produces results comparable with the combined analysis (Jones 2023).

*Biased sampling*: Many genomic data sets are subject to sampling bias (e.g. over-sampling of individuals from European ancestry, or over-sampling of individuals that have higher disease risks than the overall population). BGLR offers the possibility of including fixed effects as part of the PGS model and also can incorporate weights (currently implemented in the BGLR function or by weighting SS when using BLRCross). These capabilities can be use to account for sampling bias by either using propensity scores as fixed effects or weighting observations through inverse probability weighting.

*Prediction versus inference*: Bayesian models provide not only estimates with good statistical properties but also measures of uncertainty that fully accounts for all sources that contribute to uncertainty. The examples we present in this article are focused on PGS prediction, for this task, uncertainty quantification is useful (Ding *et al.* 2022) but often not essential. However, the methods implemented in the BLGR-R package can also be used for inferences, including estimation of heritability using both pedigree and SNPs (e.g. de los Campos, Sorensen, *et al.* 2015; Lehermeier *et al.* 2017), genetic and environmental correlations (using the Multitrait function, Pérez-Rodríguez and de los Campos 2022), and fine mapping (e.g. de los Campos *et al.* 2023; Samaddar *et al.* 2024). We refer to those studies for further details about inferences for estimation tasks commonly appearing in genetic models.

*Beyond Genomic Prediction*: The functions implemented in BGLR can take any (dense) matrices as inputs, this, together with the multiple priors available and the possibility of assigning set-specific priors, make BGLR quite flexible. While we developed BGLR with reference to genomic data, the samplers provided with it can be used to fit many kinds of generalized linear models, including models for longitudinal data, semi-parametric regressions (e.g. splines), some classes of spatial models, and models for integration of data from multiple omics (Vazquez *et al.* 2016).

### Limitations

Computations based on SS are not free of limitations. For instance, fitting models with heterogeneous error variances using SS is not straightforward; therefore, these models are implemented in BGLR but not in BLRXy or BLRCross. Likewise, models for categorical data that use data augmentation are implemented in the BGLR function but not in BLRXy or BLRCross. Therefore, for models involving those features, we only offer implementations in the BGLR function which performs computations using individual genotype–phenotype data.

Currently, the samplers included in the BGLR R-package cannot take sparse matrices as inputs. For many applications, computations can be further improved using sparse inputs, and this is an area that we aim to tackle in future updates of the package. For the PGS prediction tasks discussed in this manuscript, the use of sparse SS can lead to substantial computational improvements. However, this is an area that requires further research before such implementations are made available because (for structures that are not block-diagonal) ignoring many small LD coefficient can lead to nonpositive definite SS and highly unstable samplers. We also warn that ignoring LD between distant markers can reduce prediction accuracy if long-range LD is present due to population structure, admixture, or selection.

## Supplementary Material

jkae288_Supplementary_Data

## Data Availability

The wheat data set used in the examples is provided with the BGLR R-package. The UK-Biobank, HCHS/SOL, and All of Us data sets are publicly available through https://www.ukbiobank.ac.uk/, https://www.nimhd.nih.gov/programs/collab/HCHS-SOL/, and https://www.researchallofus.org/, respectively. Supplemental material available at G3 online.
