# Supplementary material for: Fast analysis of biobank-size data and meta-analysis using the BGLR R-package
Source: G3 (Bethesda). 2024 Dec 9;15(4):jkae288. doi: 10.1093/g3journal/jkae288 (PMC12005161; doi:10.1093/g3journal/jkae288)
Supplement: jkae288_Supplementary_Data [file jkae288_supplementary_data.pdf]

Fast Analysis of Biobank-Size Data and Meta-Analysis using the BGLR R-package

Paulino Pérez-Rodríguez[1,⋆], **Gustavo de los Campos**[2,3,4⋆], **Hao Wu**[2], **Ana I. Vazquez**[2,4], **and Kyle Jones**[2] 1: Colegio de Postgraduados, Montecillo, Estado de México 56230, México, 2: Department of Epidemiology & Biostatistics, 3: Institute for Quantitative Health Sciences and Engineering. 4: Department of Statistics & Probability, Michigan State University, East Lansing, MI 48824.

⋆: perpdgo@gmail.com and gustavoc@msu.edu.

1) Supplementary Methods

1.1) Simulating posterior samples using sufficient statistics

Using Bayes' theorem, the posterior distribution of the parameters ($\boldsymbol{\theta}$) of the models described in the main article (expressions (1) and (2), and the assumptions described when these equations are discussed) can be shown to be proportional to the product of the conditional distribution of the data given the unknowns (equation 2) times the prior, that is $p(\boldsymbol{\theta}|\boldsymbol{y}, H) \propto p(\boldsymbol{y}|\boldsymbol{\theta})p(\boldsymbol{\theta}|H)$.

In most cases, this posterior distribution does not have closed form; however, when using Gaussian (Bayesian Ridge Regression), double exponential (LASSO), Gaussian mixture (Bayes C), or Scaled t-mixture (BayesB) prior distributions for the regression coefficients and a scaled inverse chi-squared prior distributions (or inverted gamma) for the error variance, all the fully conditional distributions have closed forms (e.g, Gianola 2013; de los Campos et al. 2013). Therefore, samples can be simulated using a Gibbs sampler (Geman and Geman 1984). Because p (the number of SNP effects) is often large, sampling marker effects is the most computationally involved step of each cycle of the sampler.

The likelihood function (equation 2), and therefore the posterior distribution can be expressed either in terms of the vector of phenotypes and the matrix of genotypes $\{\boldsymbol{y}, \boldsymbol{X}\}$ or in terms of SS $\{\boldsymbol{y}'\boldsymbol{y}, \boldsymbol{X}'\boldsymbol{X}, \boldsymbol{X}'\boldsymbol{y}\}$. Sampling a single draw for a regression coefficient from its fully conditional distribution using individual genotype-phenotype data requires using operators with complexity $O(n)$; these computations must be repeated for each effect in the model, leading to an algorithm with complexity $O(np)$ per cycle of the sampler. It can be shown that the full conditional distributions for $\beta_j|else$ for the Bayesian models implemented in `BGLR` (e.g., Bayesian Ridge Regression, Bayesian LASSO, BayesA, BayesB, BayesC) is normal, with mean and variance equal to the solution (inverse of the coefficient of the left hand side) of the following equation (see de los Campos et al. 2009 for further details):

$$\left(\frac{1}{\sigma_\varepsilon^2}\boldsymbol{x}_j'\boldsymbol{x}_j + \frac{1}{\vartheta_j}\right)\beta_j = \frac{1}{\sigma_\varepsilon^2}\boldsymbol{x}_j'\boldsymbol{e}_j, \qquad (3)$$

where $\boldsymbol{x}_j$ is the j-th column of $\boldsymbol{X}$, $\boldsymbol{e}_j = \boldsymbol{y} - \boldsymbol{X}_{-j}\boldsymbol{\beta}_{-j}$, with $\boldsymbol{X}_{-j}$ the matrix $\boldsymbol{X}$ after removing the jth column, $\boldsymbol{\beta}_{-j}$ the vector $\boldsymbol{\beta}$ after removing the j-th entry, ϑ_j is a variance associated to marker j and depends on the prior assigned to marker effects. Algorithms based in this strategy do not scale well for large-n problems.

For problems with $n \geq p$ substantial improvements in computational performance can be achieved by simulating posterior samples from sufficient statistics $\{\boldsymbol{y}'\boldsymbol{y}, \boldsymbol{X}'\boldsymbol{X}, \boldsymbol{X}'\boldsymbol{y}\}$.

In this case, equation (3) can be rewritten in terms of the sufficient statistics and therefore sample $\beta_j|else$ using these inputs, that is:

$$\left(\frac{1}{\sigma_\varepsilon^2}\boldsymbol{x}_j'\boldsymbol{x}_j + \frac{1}{\vartheta_j}\right)\beta_j = \frac{1}{\sigma_\varepsilon^2}\left[\boldsymbol{x}_j'\boldsymbol{y} - (\boldsymbol{x}_j'\boldsymbol{X}\boldsymbol{\beta} - \beta_j\boldsymbol{x}_j'\boldsymbol{x}_j)\right],$$

where $\boldsymbol{x}_j'\boldsymbol{x}_j$ corresponds to the j-th diagonal element from $\boldsymbol{X'X}$, $\boldsymbol{x}_j'\boldsymbol{y}$ corresponds to the jth row from $\boldsymbol{X'y}$ and $\boldsymbol{x}_j'\boldsymbol{X}$ corresponds to the jth column from $\boldsymbol{X'X}$.

This yields an algorithm that, for each cycle, simulates posterior samples with a computational complexity $O(p)$ rather than $O(pn)$, because most of the required cross products are already pre-computed in the SS.

Computing $\boldsymbol{X'X}$ can be both memory and computationally demanding. However, the computation of $\boldsymbol{X'X}$ is an 'embarrassingly parallel' problem (Pacheco 2011); blocks of $\boldsymbol{X'X}$ can be computed separately at multiple nodes in a cluster and the blocks can then be (virtually) merged. In modern computing platforms where multicore CPU's are available, multithread optimized version of BLAS (Basic Linear Algebra Subprograms) are available and can be used to compute blocks of $\boldsymbol{X'X}$ or even the full matrix. We did not exploit this in in our study, but parallel computing is also an option that can be used to make the derivation of the SS faster. As an example, we linked R (R Core Team 2024) against OpenBLAS (https://www.openblas.net) and we re run the benchmark against `BGLR` whose results were presented in Figures 1 and 2, but now using 4 computing threads, the results are shown in Figure S2 and Table S2 and it can be seen that the computing times are improved when compared with the computing times using only one thread.

Hyper-parameters: Above we outlined the strategy used to sample regression coefficients. Once these are sampled, the hyper-parameters indexing the prior distributions of these coefficients do not involve the likelihood function. Therefore, the algorithms used to sample them are the same as the ones used in `BGLR` function which are described in Pérez and de los Campos (2014).

Sampling the error variance: Under the model described in the main article, the fully conditional distribution of the error variance depends on the residual sum of squares (RSS). Before producing any updates on regression coefficients, we set the $RSS = Var(y) \times (n-1)$, then every time a regression coefficient is updated we update the RSS using

$$RSS_{new} = RSS_{old} + \left[\beta_{j,new}^2 - \beta_{j,old}^2\right]\boldsymbol{x}_j'\boldsymbol{x}_j - 2\left[\beta_{j,new} - \beta_{j,old}\right]\left[\boldsymbol{x}_j'\boldsymbol{y} - \boldsymbol{x}_j'\boldsymbol{X}_{-j}\boldsymbol{\beta}_{-j}\right],$$

where RSS_{old} and RSS_{new} and $\beta_{j,new}$ and $\beta_{j,new}$ are the RSS and the jth coefficient before and after the update and the remaining terms are as previously defined. Note that most of the elements in the right-hand-side of the above equations are either elements or the SS or were computed when sampling the effects. Therefore, the computational burden involved in updating the RSS is minimal.

1.2) Methods used to derive Polygenic scores using hundreds of thousands of SNPs

These methods used all the available SNPs from the UK-Biobank arrays after QC (784256 SNPs). We considered four methods which are described below.

No sets: For this method, following Funkhouser et al. (2020), we split the genome into 271 overlapping segments including 3000 core SNPs (~11Mbp) plus 1000 SNPs in each of the flanking regions (~3.8 Mbp) as shown in Figure S4. We fitted models, using the `BayesB` prior using the 5000 on each segment as separate jobs in MSU's High Performance Computing Cluster (MSU-HPPC) and save the estimated effects of the core SNPs. Because we fitted separate models for each of the segments, this approach implicitly ignores LD between SNPs in the core and those outside the core and those outside of the core and the flanking regions.

To predict phenotypes in the testing set we retrieve all the estimated effects of each of the cores and computed the PGS for the testing set using the testing-set genotypes.

Sets (w/Clumping): For this method, following Yang and Zhou (2020) we classified the SNPs into a large- and small-effect sets. We identified the large effect SNPs using $p - value$ thresholding ($p - value < 5 \times 10^{-8}$)

and LD clumping (removing all SNPs with an R-sq. with leading variants greater than 0.1). The small-effect set consisted on all the other SNPs. Then, we fitted a model of the following form

$$\boldsymbol{y} = \mathbf{1}\mu + \boldsymbol{X}_1\boldsymbol{\beta}_1 + \boldsymbol{X}_{2,q}\boldsymbol{\beta}_{2,q} + \boldsymbol{\varepsilon},$$

for each of the 271 segments. Above, $\boldsymbol{X}_1$ included the large-effect SNPs and $\boldsymbol{X}_{2,q}$ included all the small-effect SNPs in the qth ($q = 1, \ldots, 271$) chromosome segment (including those in the core and those on the two flanking regions). For both sets we used the `BayesB` prior; however, to avoid over-shrinking large-effect SNPs we assigned (and estimated through posterior sampling) different hyper-parameters (the proportion of non-zero effects and the scale parameter of the scaled$-t$ slab, *df* was fixed at 5) for each of the sets. From each run, we saved the estimated posterior mean of the large-effect SNPs and the estimated effects of the core of the qth segment.

To derive prediction, we average the estimated effects for the large effect SNPs from each of the 271 runs and retrieve the estimated effects for the small-effect SNP set from each of the cores and computed the PGS for the testing set using the testing-set genotypes.

Sets (No Clumping): This method is similar to *Sets (w/Clumping)* with the only difference that we did not applied the clumping step to prune the large-effect SNP set. Therefore, there many more SNPs included in $\mathbf{X}_1$ in each of the runs.

Sets (2 Steps PGS): This method uses the same SNP sets as *Sets (w/Clumping)*; however, instead of fitting the model above described, in a first step we estimated the large-effects fitting a model of the form $\boldsymbol{y} = \mathbf{1}\mu + \boldsymbol{X}_1\boldsymbol{\beta}_1 + \boldsymbol{\varepsilon}$, then computed a PGS for the training set using $\boldsymbol{PGS}_1 = \boldsymbol{X}_1\hat{\boldsymbol{\beta}}_1$, and, in a second set we estimated the the small-effect SNP effects using a model of the form

$$\boldsymbol{y} = \mathbf{1}\mu + \boldsymbol{PGS}_1\alpha + \boldsymbol{X}_{2,q}\boldsymbol{\beta}_{2,q} + \boldsymbol{\varepsilon},$$

where α is the (fixed) effect of the PGS derived in the first step, and $\boldsymbol{X}_{2,q}$ are all the SNPs in segment q, except those that were part of the large-effect SNP set.

To derive predictions for the testing set we retrieve the SNP effects from both sets estimated in steps 1 and 2, and computed PGS for the testing genotypes in the usual manner.

The number of SNPs entering in the large- and small-effect sets for each of the methods and traits is presented in Table S4.

2) Supplementary Figures

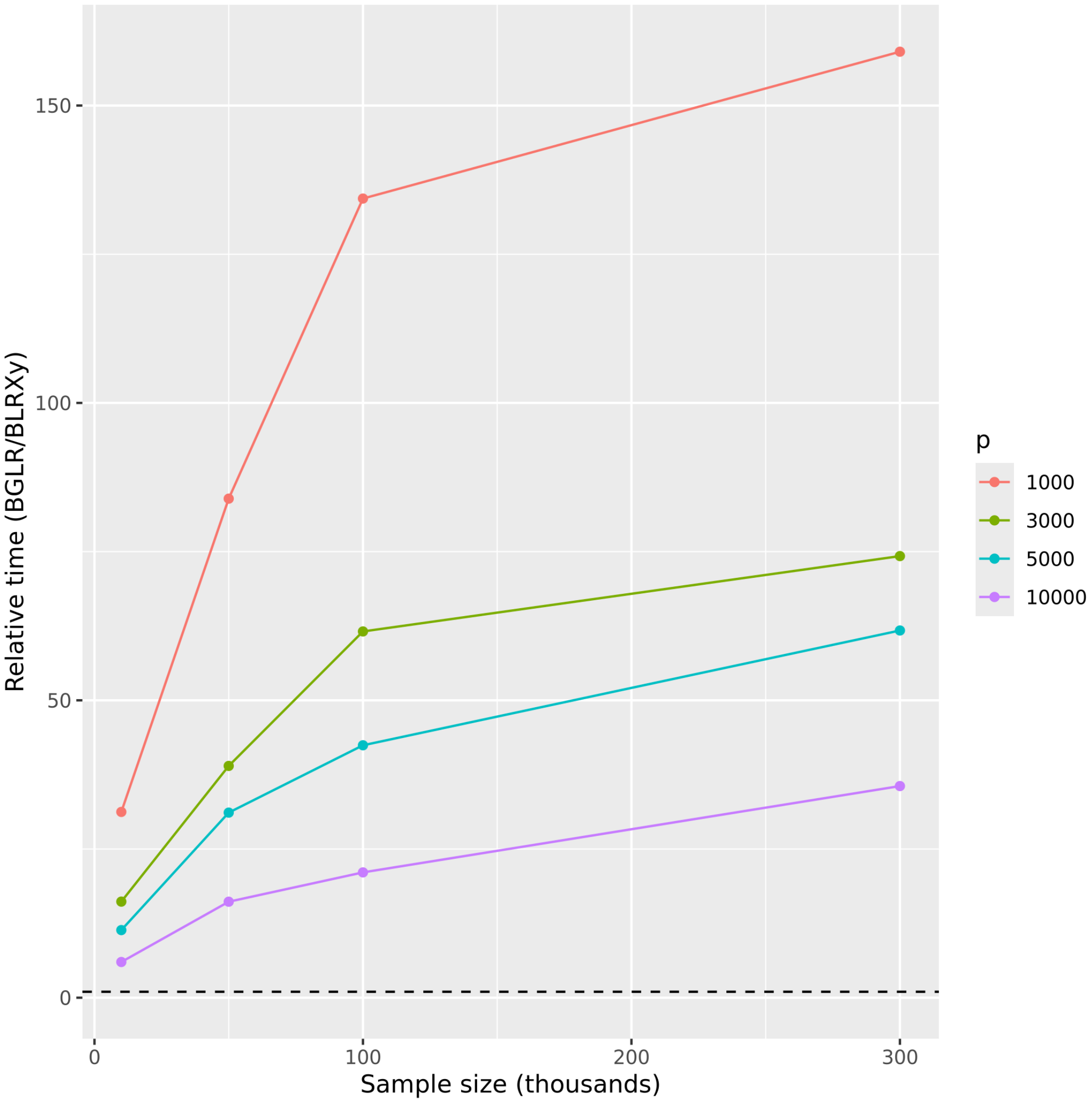

Figure S1: Computational time used by the `BLRXy` function to generate 3000 posterior samples, relative to the computational time used by the `BGLR` function to simulate the same number of samples, by sample size and the number of predictors (SNPs) in the model. The prior used was **BayesC** and the computations were done using a **single thread**. The simulation settings are described in the Benchmark 1 section of the manuscript. See Figure 1 and Table S1 for actual times.

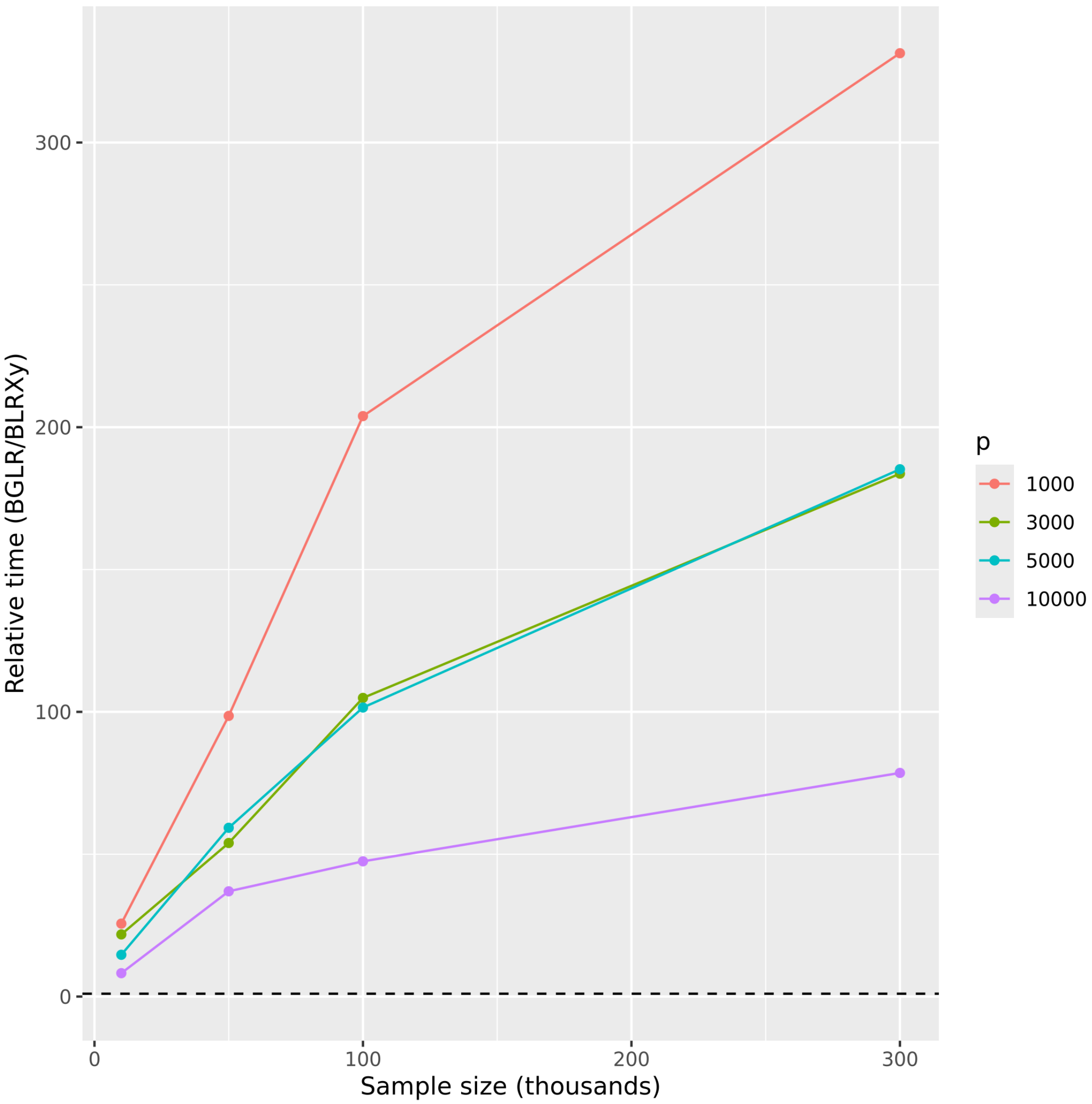

Figure S2: Computational time used by the BLRXy function to generate 3000 posterior samples, relative to the computational time used by the BGLR function to simulate the same number of posterior samples when using **four threads**. The simulation settings are described in the section Benchmark 1 of the manuscript, and the prior used was **BayesC**. See Table S2 for actual times.

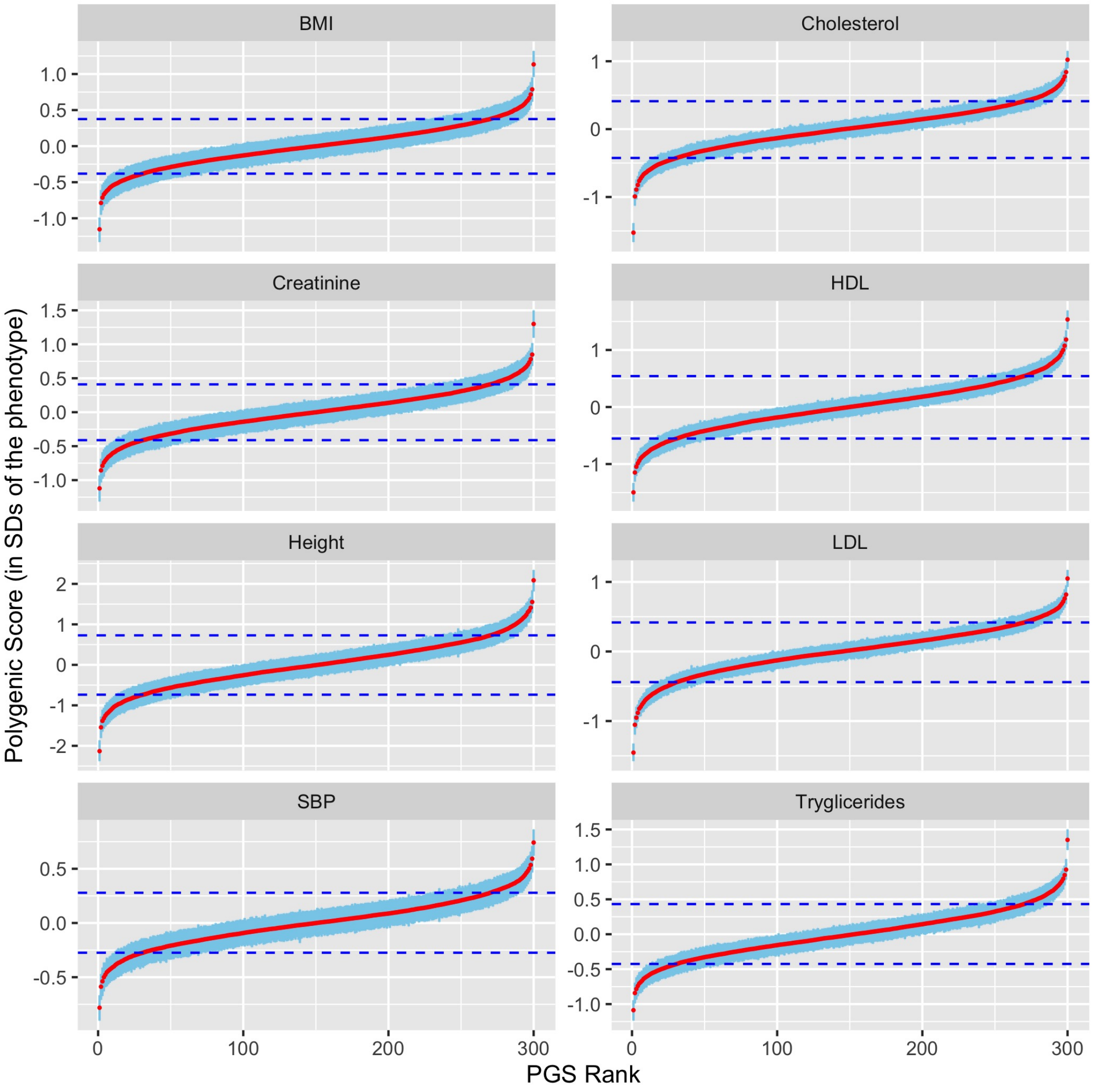

Figure S3: Polygenic Score (red) and 95% Credibility Interval (skyblue), derived using SNPs with GWAS $p-value < 1 \times 10^{-5}$ (Model BayesB, see Figure 3 for the accuracy of these PGS), expresed as deviations from the trait mean and in SDs of the trait. Each point represents a subject, the PGSs of only 300 randomly selected subjects are displayed. The horizontal dashed lines mark the 10th and 90th empirical percentiles of the PGS, respectively.

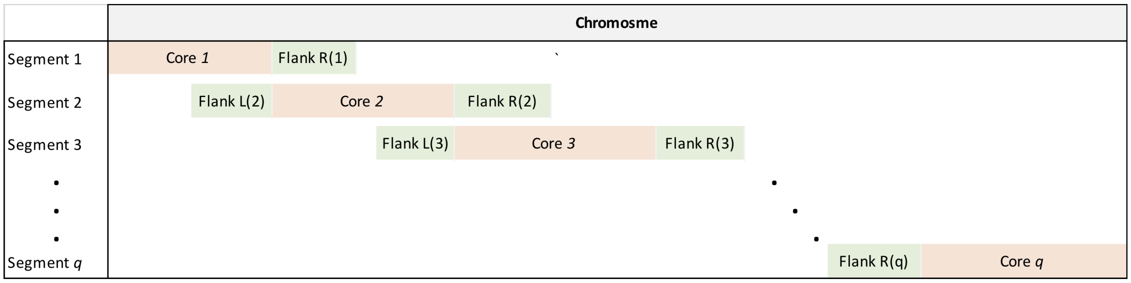

Figure S4: Graphical representation of the partition of chromosome into overlaping segments. In our application cores included 3000 SNPs ($\sim$ 11Mbp) and each of the flanking regions 1000 SNPs ($\sim$ 3.8Mbp).

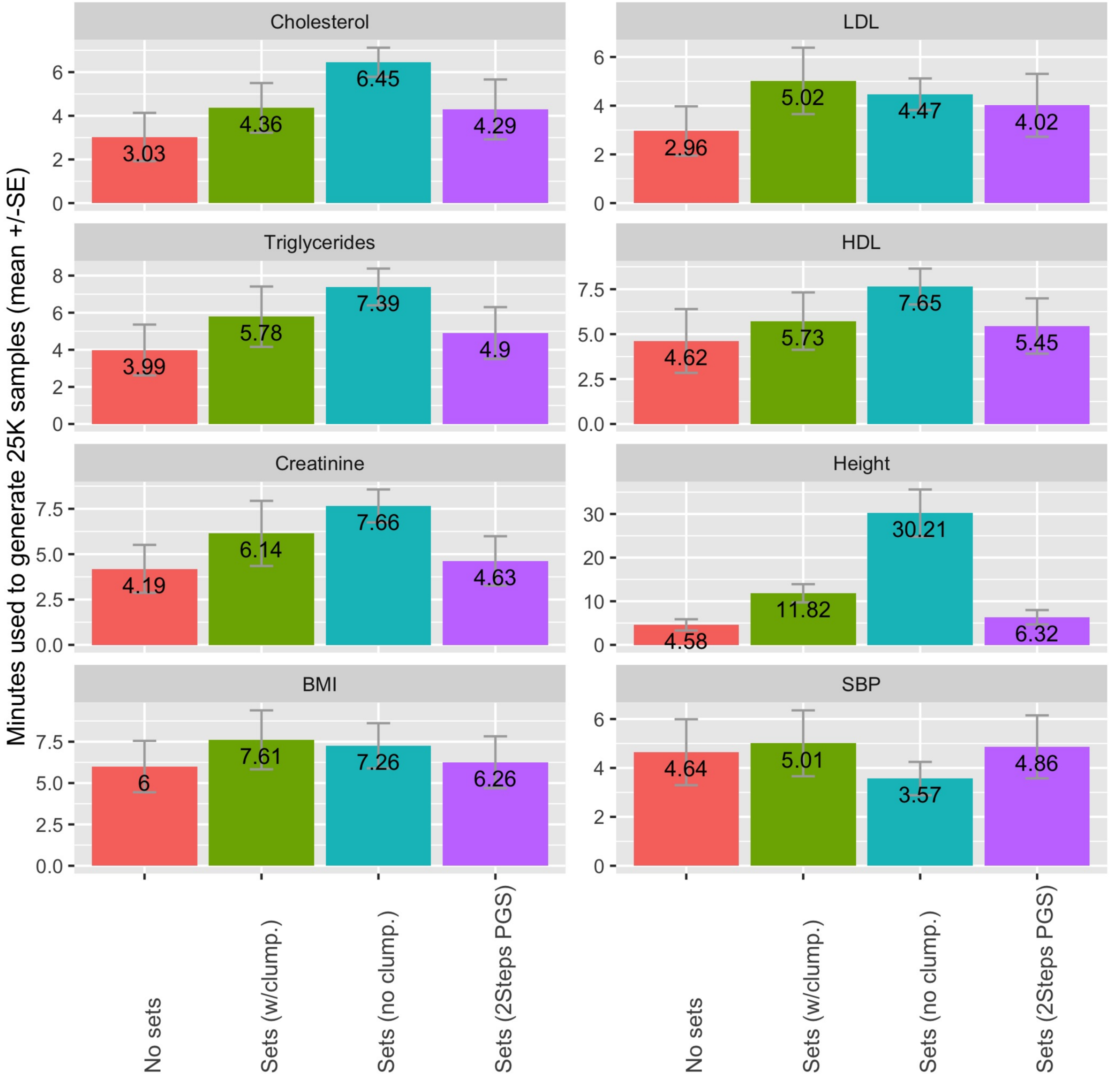

Figure S5: Computing time (in minutes) required to simulate 25000 posterior sample for a chromosome segment (5000 SNPs plus the large-effect SNPs) by trait and method. Models were run using **four threads** and the prior used was **BayesB**.

3) Supplementary Tables

Table S1: Computing times (in seconds) for crossproducts, sampling and fitting linear model with founctions `BLRXy` and `BGLR`. The simulation settings are described in the section Benchmark 1 of the manuscript. The prior used was **BayesC** and all computations were done using **one thread**.

n	p	Time crossproduct	SE	Time sampling	SE	Time BLRXy	SE	Time BGLR	SE
10000	1000	0.16	0.00	0.55	0.01	0.71	0.01	22.26	0.53
10000	3000	1.25	0.05	2.70	0.10	3.94	0.14	63.70	1.34
10000	5000	3.39	0.04	7.07	0.40	10.46	0.41	118.88	3.64
10000	10000	14.53	0.59	24.38	2.23	38.91	2.51	233.58	3.46
50000	1000	0.79	0.01	0.52	0.00	1.31	0.01	109.65	2.07
50000	3000	5.96	0.19	2.00	0.07	7.96	0.21	310.19	6.32
50000	5000	14.52	0.48	3.68	0.28	18.20	0.57	566.40	17.95
50000	10000	65.52	2.83	11.80	0.80	77.33	3.07	1248.15	60.31
100000	1000	1.53	0.02	0.52	0.01	2.06	0.02	276.58	3.52
100000	3000	11.99	0.38	1.85	0.05	13.85	0.34	852.74	23.56
100000	5000	30.47	0.29	3.65	0.06	34.13	0.25	1448.36	28.43
100000	10000	133.37	5.75	12.50	0.83	145.87	5.48	3073.48	21.57
300000	1000	5.35	0.47	0.59	0.02	5.94	0.41	944.44	25.35
300000	3000	36.59	1.17	2.12	0.08	38.71	1.02	2873.94	80.10
300000	5000	108.89	10.99	4.13	0.20	113.02	9.15	6977.69	367.67
300000	10000	386.03	34.26	11.29	0.55	397.32	28.30	14138.81	148.69

Table S2: Computing times (in seconds) for crossproducts, sampling and fitting linear model with function `BLRXy`. The simulation settings are described in the section Benchmark 1 of the manuscript. The prior used was **BayesC** and all computations were done using **four threads**, for the correspondig times with **one thread** for `BGLR`, see Table S1.

n	p	Time crossproduct	SE	Time sampling	SE	Time BLRXy	SE
10000	1000	0.10	0.01	0.77	0.04	0.87	0.04
10000	3000	0.42	0.01	2.50	0.10	2.92	0.11
10000	5000	1.47	0.04	6.64	0.31	8.10	0.32
10000	10000	5.22	0.14	23.22	1.42	28.44	1.43
50000	1000	0.42	0.02	0.69	0.03	1.11	0.04
50000	3000	3.25	0.29	2.50	0.11	5.75	0.28
50000	5000	5.67	0.13	3.89	0.17	9.55	0.21
50000	10000	23.22	0.80	10.54	0.36	33.76	0.74
100000	1000	0.74	0.04	0.62	0.03	1.36	0.07
100000	3000	6.03	0.48	2.10	0.05	8.13	0.41
100000	5000	10.60	0.30	3.66	0.10	14.27	0.29
100000	10000	53.32	4.15	11.38	0.72	64.70	3.89
300000	1000	2.20	0.14	0.65	0.03	2.85	0.15
300000	3000	13.55	0.31	2.10	0.04	15.65	0.28
300000	5000	33.32	1.00	4.35	0.21	37.67	0.90
300000	10000	163.66	12.45	16.31	0.95	179.97	10.83

Table S3: Computing times (in seconds) for crossproducts, sampling and fitting linear model with founctions `BLRXy` and `BGLR`. The simulation settings are described in the section Benchmark 1 of the manuscript. The prior used was **BRR** and all computations were done using **one thread**.

n	p	Time crossproduct	SE	Time sampling	SE	Time BLRXy	SE	Time BGLR	SE
10000	1000	0.18	0.01	1.50	0.05	1.69	0.06	35.47	0.59
10000	3000	1.07	0.02	16.70	0.10	17.77	0.11	105.36	1.83
10000	5000	3.40	0.11	46.84	0.21	50.24	0.23	209.17	6.16
10000	10000	12.12	0.28	191.87	2.40	203.99	2.46	347.91	19.34
50000	1000	0.70	0.01	1.34	0.00	2.04	0.01	237.69	2.52
50000	3000	5.06	0.04	16.36	0.10	21.42	0.13	708.55	2.37
50000	5000	13.99	0.14	48.19	0.78	62.18	0.89	1361.57	42.66
50000	10000	54.33	0.84	189.43	1.83	243.76	2.38	2461.45	19.73
100000	1000	1.52	0.05	1.58	0.08	3.10	0.12	473.45	4.49
100000	3000	10.51	0.07	16.47	0.10	26.98	0.16	1546.32	30.10
100000	5000	28.43	0.37	50.00	0.99	78.44	1.28	2451.81	8.25
100000	10000	109.14	1.51	190.39	1.81	299.54	2.89	5629.80	169.28
300000	1000	4.42	0.08	1.49	0.06	5.91	0.13	1480.44	14.97
300000	3000	31.68	0.18	16.57	0.10	48.24	0.25	4489.54	35.74
300000	5000	85.74	1.05	48.53	0.79	134.27	1.63	8566.70	151.84
300000	10000	336.57	1.63	273.99	8.49	610.56	7.98	21600.06	121.60

Table S4: Number of SNPs used in Whole-Genome PGS by trait, set, and clumping strategy. See Supplementary Methods, section 1.2 for details about the methodology used to derive these PGS.

Trait	Clumping	Large Effects	Small Effects
BMI	Yes (R2>0.1)	375	783881
Cholesterol	Yes (R2>0.1)	385	783871
Creatinine (log)	Yes (R2>0.1)	482	783774
HDL	Yes (R2>0.1)	539	783717
Height	Yes (R2>0.1)	1945	782311
LDL	Yes (R2>0.1)	339	783917
SBP	Yes (R2>0.1)	183	784073
Triglycerides	Yes (R2>0.1)	412	783844
BMI	No	1733	782523
Cholesterol	No	2758	781498
Creatinine (log)	No	2545	781711
HDL	No	2875	781381
Height	No	10891	773365
LDL	No	1959	782297
SBP	No	747	783509
Triglycerides	No	2943	781313

Table S5: Sample size of individuals of Hispanic ancestry from the All of Us (AOU) and the HCSL/SOL cohorts used by trait and data set.

Trait	AOU (Hispanic)	HCSL/SOL (Hispanic)
Height	56784	12386
BMI	56004	12368
Cholesterol	19598	12394
SBP	57902	12397
HDL	18998	12393
LDL	11432	12135
Triglycerides	19354	12394
Log-Creatinine	31954	12394

Table S6: Average correlation (and % change relative to within HCSL/SOL prediction) by trait and training data.

Trait	Training data	Sq. Correlation	% change
SBP	SOL	0.010	–
SBP	AOU	0.015	51.27%
SBP	UKB	0.015	50.46%
SBP	SOL+AOU	0.017	71.70%
SBP	SOL+UKB	0.018	85.51%
SBP	AOU+UKB	0.019	93.38%
SBP	SOL+AOU+UKB	0.020	99.02%
BMI	SOL	0.020	–
BMI	AOU	0.040	101.34%
BMI	UKB	0.042	106.48%

Trait	Training data	Sq. Correlation	% change
BMI	SOL+AOU	0.043	115.26%
BMI	SOL+UKB	0.045	122.57%
BMI	AOU+UKB	0.049	141.87%
BMI	SOL+AOU+UKB	0.050	151.04%
Creatinine	SOL	0.027	–
Creatinine	AOU	0.029	6.55%
Creatinine	UKB	0.041	51.61%
Creatinine	SOL+AOU	0.034	26.87%
Creatinine	SOL+UKB	0.051	86.19%
Creatinine	AOU+UKB	0.054	97.51%
Creatinine	SOL+AOU+UKB	0.055	103.01%
Triglycerides	SOL	0.057	–
Triglycerides	AOU	0.064	12.30%
Triglycerides	UKB	0.070	23.30%
Triglycerides	SOL+AOU	0.069	21.40%
Triglycerides	SOL+UKB	0.076	33.06%
Triglycerides	AOU+UKB	0.078	36.13%
Triglycerides	SOL+AOU+UKB	0.080	39.26%
Cholesterol	SOL	0.068	–
Cholesterol	AOU	0.062	-8.89%
Cholesterol	UKB	0.074	8.37%
Cholesterol	SOL+AOU	0.080	17.66%
Cholesterol	SOL+UKB	0.080	17.70%
Cholesterol	AOU+UKB	0.080	18.43%
Cholesterol	SOL+AOU+UKB	0.085	25.36%
LDL	SOL	0.077	–
LDL	AOU	0.046	-40.55%
LDL	UKB	0.079	2.58%
LDL	SOL+AOU	0.075	-2.72%
LDL	SOL+UKB	0.086	12.39%
LDL	AOU+UKB	0.084	8.56%
LDL	SOL+AOU+UKB	0.088	14.85%
HDL	SOL	0.098	–
HDL	AOU	0.107	8.16%
HDL	UKB	0.116	18.15%
HDL	SOL+AOU	0.118	20.06%
HDL	SOL+UKB	0.126	27.72%
HDL	AOU+UKB	0.129	30.52%
HDL	SOL+AOU+UKB	0.132	33.92%
Height	SOL	0.157	–
Height	AOU	0.216	37.84%
Height	UKB	0.185	17.56%
Height	SOL+AOU	0.228	45.33%
Height	SOL+UKB	0.229	45.97%
Height	AOU+UKB	0.248	58.18%
Height	SOL+AOU+UKB	0.254	61.59%
Average	SOL	0.056	–
Average	AOU	0.063	11.74%
Average	UKB	0.070	25.06%
Average	SOL+AOU	0.073	30.80%
Average	SOL+UKB	0.080	42.24%
Average	AOU+UKB	0.083	47.45%

Trait	Training data	Sq. Correlation	% change
Average	SOL+AOU+UKB	0.085	52.49%

4) Supplementary scripts

4.1) A small example demonstrating how to fit models using sufficient statistics from multiple sources

The script provided below gives a simple demonstration using a data set with 599 wheat lines and 500 SNPs. In the example, we first fit the model to the combined data set using `BGLR`. Then, we split the data set into two subsets, derive the SS for each set, combine the SS, and fit the model to the combined SS using `BLRCross`, apart from a small MC error, the parameter estimates, and the posterior distributions are identical.

```
X<-scale(wheat.X[,1:500],center=TRUE,scale=TRUE)
y<-wheat.Y[,1]

#For a reference, we fit the model to the
#combined data
fm4<-BLRXy(y=y,ETA=list(list(X=X,model="BRR")),
    nIter=12000,burnIn=2000)

#Here we split the data into two data sets at random
set.seed(195021)
set<-sample(1:2,size=nrow(X),replace=TRUE)

X1<-X[set==1,]; y1<-y[set==1]
X2<-X[set==2,]; y2<-y[set==2]

#Next, we compute the sufficient statistics (SS)
#by data set
XX1<-crossprod(X1); Xy1<-crossprod(X1,y1)
n1<-nrow(X1); m1<-mean(y1); v1<-var(y1)
XX2<-crossprod(X2); Xy2<-crossprod(X2,y2)
n2<-nrow(X2); m2<-mean(y2); v2<-var(y2)

#Then, we combine the SS
XX<-XX1+XX2
Xy<-Xy1+Xy2
n<-n1+n2
w1<-n1/(n1+n2); w2<-1-w1
mY<-m1*w1+m2*w2
vY<-v1*w1+v2*w2

#Then, we fit the model
fm5<-BLRCross(n=n,my=mY,vy=vY,Xy=Xy,XX=XX,
    idPriors = rep(1,ncol(XX)),
    priors=list(list(model="BRR")),
    nIter=12000,burnIn=2000)

cor(fm4$ETA[[1]]$b,fm5$ETA[[1]]$b)
```

4.2) Pipeline

The scripts used for benchmarks and data analysis are provided in the file **SCRIPTS.zip** which contains folders and subfolders per benchmark/analysis.

References

de los Campos, G., J. M. Hickey, R. Pong-Wong, H. D. Daetwyler, and M. P. L. Calus. 2013. "Whole Genome Regression and Prediction Methods Applied to Plant and Animal Breeding." *Genetics* 193: 327–45. https://doi.org/10.1534/genetics.112.143313.

de los Campos, G., H. Naya, D. Gianola, J. Crossa, A. Legarra, E. Manfredi, K. Weigel, and J. M. Cotes. 2009. "Predicting Quantitative Traits with Regression Models for Dense Molecular Markers and Pedigree." *Genetics* 182 (1): 375–85.

Funkhouser, S. A., A. I. Vazquez, J. P. Steibel, C. W. Ernst, and G. de los Campos. 2020. "Deciphering Sex-Specific Genetic Architectures Using Local Bayesian Regressions." *Genetics* 215 (1): 231–41.

Geman, S., and D. Geman. 1984. "Stochastic Relaxation, Gibbs Distributions and the Bayesian Restoration of Images." *IEEE Transactions on Pattern Analysis and Machine Intelligence* 6 (6): 721–41.

Gianola, D. 2013. "Priors in Whole-Genome Regression: The Bayesian Alphabet Returns." *Genetics* 90 (December): 525–40.

Pacheco, P. 2011. *An Introduction to Parallel Programming.* 1st ed. San Francisco, CA, USA: Morgan Kaufmann Publishers Inc.

Pérez, Paulino, and Gustavo de los Campos. 2014. "Genome-Wide Regression and Prediction with the BGLR Statistical Package." *Genetics* 198 (2): 483–95. https://doi.org/10.1534/genetics.114.164442.

R Core Team. 2024. *R: A Language and Environment for Statistical Computing.* Vienna, Austria: R Foundation for Statistical Computing. https://www.R-project.org/.

Yang, Sheng, and Xiang Zhou. 2020. "Accurate and Scalable Construction of Polygenic Scores in Large Biobank Data Sets." *The American Journal of Human Genetics* 106 (5): 679–93. https://doi.org/https://doi.org/10.1016/j.ajhg.2020.03.013.
